# PReS-FINAL-2366: Paediatric rheumatology in South Africa

**DOI:** 10.1186/1546-0096-11-S2-O31

**Published:** 2013-12-05

**Authors:** C Scott

**Affiliations:** 1 Paediatric Rheumatology, Red Cross War Memorial Children's Hospital, Cape Town, South Africa

## Introduction

South Africa is a middle income, developing country with a population of 51 million people, 29% of whom are younger than 14 years. As a result of a heavy burden of communicable disease and major socio-economic and political challenges, chronic rheumatic conditions in children have received very little attention in the past and South Africa only has 5 pediatric rheumatologists, 1 for every 3 million children.

## Objectives

1) To describe the past and present of pediatric rheumatology in South Africa. 2) To describe challenges in the care of PR patients in South Africa. 3) To describe training and research activities in South Africa and speculate on the future.

## Methods

Available regional pediatric rheumatology data, clinic data and pediatric rheumatology workforce statistics were reviewed.

## Results

The spectrum of rheumatic diseases seen in South African clinics is different to those in developed countries. The burden of infectious diseases complicates the presentation and management of children with rheumatic diseases. There is a shortage of pediatric rheumatologists and training opportunities in South Africa.

## Conclusion

In recent years Pediatric Rheumatology has become an established discipline in South Africa and the provision of specialized healthcare for children and adolescents with Rheumatic Diseases has improved. Despite this the majority of children in South Africa are not able to access appropriate care. There is a need for training in pediatric rheumatology at all levels of pediatric healthcare in South Africa and a critical need to support the training and retention of pediatric rheumatologists. There is a shortage of research on pediatric rheumatic diseases in Africa.

## Disclosure of interest

None declared.

